# Modulation of Bone and Joint Biomarkers, Gut Microbiota, and Inflammation Status by Synbiotic Supplementation and Weight-Bearing Exercise: Human Study Protocol for a Randomized Controlled Trial

**DOI:** 10.2196/30131

**Published:** 2021-10-26

**Authors:** Bolaji Lilian Ilesanmi-Oyelere, Nicole C Roy, Marlena C Kruger

**Affiliations:** 1 College of Health Massey University Palmerston North New Zealand; 2 Department of Human Nutrition University of Otago Dunedin New Zealand

**Keywords:** synbiotic (prebiotic+probiotic), weight-bearing exercise, gut microbiota, inflammation, BMD, cytokines, bone and joint biomarkers

## Abstract

**Background:**

There is strong evidence suggesting that prebiotics and probiotics regulate gut microbiota, reducing inflammation and thereby potentially improving bone health status. Similarly, mechanistic evidence suggests that either low-impact or high-impact weight-bearing exercises improve body composition and consequently increase bone mineral density in individuals with osteoporosis and osteoarthritis.

**Objective:**

This study aims to investigate the effects of a synbiotic (probiotic+prebiotic) supplementation, an exercise intervention, or a combination of both on gut microbiota, inflammation, and bone biomarkers in postmenopausal women.

**Methods:**

A total of 160 postmenopausal women from New Zealand will be recruited and randomized to one of four interventions or treatments for 12 weeks: control, synbiotic supplementation, exercise intervention, or synbiotic supplementation and exercise. The primary outcome measure is the bone and joint biomarkers at baseline and week 12, whereas the gut microbiota profile and inflammatory cytokine measurements will serve as the secondary outcome measures at baseline and week 12. Baseline data and exercise history will be used to assess, allocate, and stratify participants into treatment measures.

**Results:**

Recruitment of participants will begin in September 2021, and the anticipated completion date is June 2022.

**Conclusions:**

To the best of our knowledge, this will be the first randomized controlled trial to analyze the effects of both a synbiotic supplement and an exercise intervention in postmenopausal women. On the basis of the results obtained, a combination of synbiotic supplements and exercise might serve as a noninvasive approach to manage and/or improve body composition and bone health in postmenopausal women.

**Trial Registration:**

Australian New Zealand Clinical Trials Registry ACTRN12620000998943p; https://www.anzctr.org.au/Trial/Registration/TrialReview.aspx?id=380336&isClinicalTrial=False

## Introduction

### Background

The global population is aging. The global incidence of postmenopausal osteoporosis is also increasing [1,2]. Postmenopausal osteoporosis is characterized by increased low-grade inflammation that contributes to low bone mass and degradation of bone mineral content, resulting in postmenopausal bone loss [3-5]. Elevated levels of proinflammatory cytokines (eg, interleukin [IL]–6, tumor necrosis factor [TNF]-α, IL-1, and receptor activator of nuclear factor kappa-Β ligand produced by activated T cells) induce osteoclast formation and activity during senescence [2,6]. Meanwhile, it is well recognized that conventional estrogen therapy in the form of hormone replacement therapy (HRT), such as estradiol implants, increases bone density by increasing the activity of osteoblasts, reducing bone resorption, and reducing inflammation [7-9]. However, the long-term use of HRT as an anabolic treatment and high doses of estrogen may not reduce the incidence of bone fracture [7] and are associated with long-term side effects. Therefore, nutritional interventions are safe and reliable for improving bone health status.

Studies have reported that changes in the gut microbiome can affect distant organs, including the bone and subsequently the development of osteoporosis [10]. Recent studies in rats and mice have suggested that the gut microbiome modulates immune status [11] as well as calcium absorption and molecular control of bone resorption [12-14]. Although studies have been conducted in animals, there are few studies in humans that have investigated the effects of pre- and probiotics on the gut microbiome and postmenopausal osteoporosis. Moreover, human gut microbiota is different from that of rodents, which is why many studies have faced limited success in their attempt to *humanize* the murine microbiota [15].

### Defining Osteoporosis and Its Significance

Osteoporosis is “characterized by low bone mass and microarchitectural deterioration of bone tissues, consequently increasing bone fragility and breakage” [16]. The term osteoporosis (*osteoun*) refers to *bone* and *pór* (*os*) to *passage+osis* [17]. The widely accepted clinical diagnostic criteria and intervention threshold are defined as bone mineral density (BMD) ≥2.5 SDs (T score ≤−2.5) below the mean value of a young reference at the lumbar spine, femur neck, or total hip bone in older men and postmenopausal women [18].

The vast burden of osteoporosis constitutes an increase in morbidity and mortality [19], loss of quality-adjusted life-years (QALYs) [20], and a continuous rise in the cost of health care services, such as clinics, nursing homes, and hospitals [21,22]. It is a growing global health concern, with the lifetime risk of sustaining any fracture at approximately 50% for women and 20% for men in individuals aged >50 years living in Western countries [23]. It has also been predicted to become an issue in developing countries as the aging population rises. The risk of fracture becomes higher as people age, especially in postmenopausal women [24].

In 2011, osteoporosis-related QALYs were found to be 11,249, with a projection of 13,205 and 15,176 for 2013 and 2020, respectively, because of fractures. The results also suggest that there are more QALYs for women (6028) than men (5221) [22].

As a global epidemic situation, the International Osteoporosis Foundation suggests that 33% of middle-aged women and 20% of men (aged >50 years) will have an osteoporotic fracture [25]. The disease constitutes an economic burden, particularly because of the high cost of treatment. Over US $300 million per annum is estimated to be spent on treating fractures, whereas the total cost is estimated at US $1.15 billion per annum in health costs, posing a heavy burden on health care service providers in New Zealand [20].

### Physical Activity

Physical activity is defined by the World Health Organization as any “bodily movement of the skeletal muscle, which requires energy expenditure” [26]. Physical activity enhances the composition and function of the human skeletal system. However, skeletal muscle performance also deteriorates with age [27], and a lack of exercise and physical activity has been linked to bone loss and ultimately osteoporosis [28]. Osteoporosis may cause falls that result in osteoporotic fractures in older women.

Previous studies, including Cochrane reviews, have reported the effects of physical activity on bone strength across the life spans [29] of children and adolescents [30] as well as women, especially postmenopausal women [31-33]. A study from Italy reported progressive resistance strength training of the lower limbs to be most effective for the neck of the femur BMD in patients with osteoporosis. In contrast, a multicomponent training program was suggested for a spine BMD intervention [34].

Neilson et al [35] have defined activity energy expenditure (AEE) as “a modifiable component of total energy expenditure (TEE) derived from both volitional and non-volitional activities.“ Total energy expenditure comprises multiple components, including physical activity energy expenditure, resting energy expenditure, and the thermic effect of food [36]. The impact of physical activity has been used to alleviate several obesity-related diseases and the overall burden of diseases in men and women alike [37].

### The Research

#### Overview

The gut microbiome could be a plausible target for the modulation of bone disorders in the aged, as it has been associated with the innate and adaptive immune system. Our previous study inquired into the association between the gut microbiome and bone health status based on the World Health Organization classification of osteoporosis among postmenopausal women [38]. The relationship between the composition and predictive function of gut microbiota in women and their bone density, classified into healthy and osteopenic or osteoporotic groups, was investigated. The findings of this recent study showed that α diversity of the microbial profiles differed based on the hip and femoral neck osteoporosis classifications. Meanwhile, β diversity principal component analysis by using the Bray-Curtis index showed differences based on femoral neck classifications. Positive correlations were observed between *Lactobacillus, Bacillus, Paenibacillus*, and *Geobacillus* (all from the phylum Firmicutes) and BMD at all sites. However, negative correlations were reported for *Bacteroides* and *Parabacteroides* and all BMD sites [39,40]. This finding agrees with previous reports that showed the importance of the *Lactobacillus* species in bone maintenance [14,41]. This result was similar to that of Li et al [42], who showed a negative correlation between *Bacteroides* and BMD.

In addition, a study conducted with ovariectomized rats showed the effects of probiotics, prebiotics, and synbiotics on mineral (calcium and phosphorus) metabolism and absorption. The effects were also observed in the form of higher *Bifidobacteria* and *Bacteroides* counts, lower pH, and reduced bone turnover. The intervention also resulted in a tendency toward lower bone alkaline phosphatase [43].

Therefore, modulation with a synbiotic food supplement and weight-bearing low-impact exercise (interventions) can be used to provide data that may contribute to improvements in osteoporotic patient care. This study will use a prospective stratified (by exercise history), randomized, 4-group experimental design with 2 major data collection points (baseline and week 12). Before randomization, participants will be stratified by exercise history (≥2 high-intensity exercise sessions per week and <2 sessions per week) using the International Physical Activity Questionnaire to ensure equal distribution among the 4 groups. However, all participants would be permitted to continue their usual physical activity regime.

#### Research Questions

**Figure 1:**
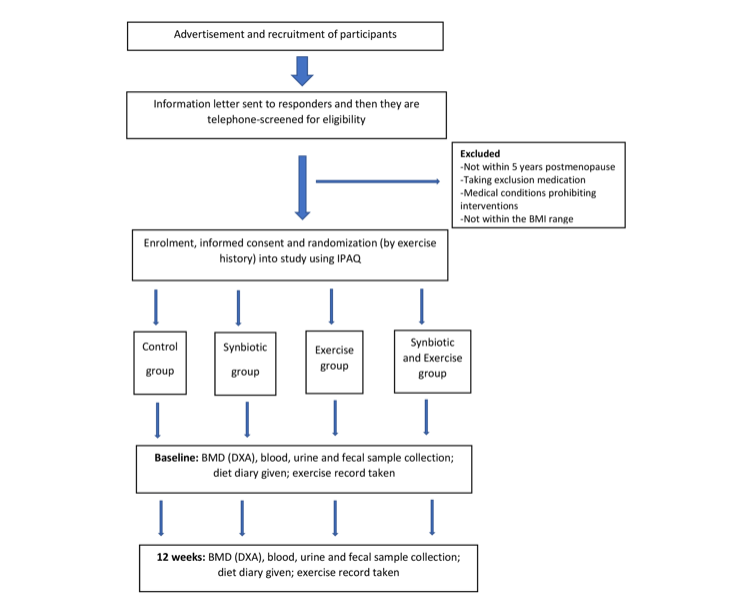
Cartilage oligomeric matrix protein 4 Bones clinical study flowchart. BMD: bone mineral density; DXA: dual-energy x-ray absorptiometry; IPAQ: International Physical Activity Questionnaire.

What are the effects of dietary interventions with synbiotic food supplements and weight-bearing exercises on bone metabolism, gut microbiota, and micronutrient or inflammation status in postmenopausal women? Do specific gut microbiota alterations moderate bone metabolism? How do individual differences in nutritional interventions affect gut microbiota and, subsequently, bone metabolism? Ultimately, can they be used as a treatment for postmenopausal osteoporosis? Our principal hypothesis is that improvement in bone health, gut microbiota, and inflammation status will be greater in participants randomized to the synbiotic+exercise group compared with participants in the control, synbiotic, or the exercise groups (Figure 1).

#### Specific Aim

This study aims to examine whether supplementation of fermented milk or dairy (yogurt) with a synbiotic (probiotic+prebiotic) and weight-bearing low-impact exercise could be effective in achieving favorable changes in gut microbiota, inflammation status, and biochemical indexes of bone and joint metabolism.

Aim 1: to compare control, synbiotic, exercise, and synbiotic+exercise groups based on changes in the gut microbiota using 16S rDNA sequencing at baseline and week 12.

Aim 2: to compare control, synbiotic, exercise, and synbiotic+exercise groups based on changes in inflammation status (inflammatory cytokines) at baseline and week 12.

Aim 3: to compare control, synbiotic, exercise, and synbiotic+exercise groups based on changes in bone formation (procollagen type 1 N-terminal propeptide [P1NP]), resorption (cross-linked C-telopeptide of type 1 collagen [CTx-I]), and joint degradation (CTx-II/COMP [cartilage oligomeric matrix protein]) at baseline and week 12.

Aim 4: to compare control, synbiotic, exercise, and synbiotic+exercise groups based on changes in body composition (lean body mass and fat mass), total hip, femoral neck, and spine BMD (DXA [dual-energy x-ray absorptiometry]) at baseline and week 12.

## Methods

### Participants

The G*Power statistical software, version 3.1.9.7, developed in Heinrich Heine University Düsseldorf, was used to calculate the sample size using the bone biomarker CTx-I; a recommended number of 36 women would be required for the study. However, 40 women aged >60 years will be recruited for each of the 4 groups to allow for a possible dropout rate of 10%. The test groups will receive the synbiotic food supplementation and exercise program (10,000 steps brisk walking per day required), whereas the control group will receive a placebo and no exercise; however, dietary intake and exercise will be monitored by a 3-day diet diary and the International Physical Activity Questionnaire. All study participants will read the information sheet, and signed and written consent forms will be obtained from them.

### Inclusion Criteria

The inclusion criteria include a confirmed menopause diagnosis (by an initial blood test—baseline screening—that includes checking the levels of follicle-stimulating hormone [≥30 mIU/mL] and estrogen) of at least five years based on no menstruation and the BMI of all participants will be between 17 and 35 kg/m².

### Exclusion Criteria

The following criteria are to be confirmed by medical history or measurements:

1. Use of HRT
2. Biphosphonates in the past 6 months
3. Currently on estrogen, tamoxifen, aromatase inhibitors, or other antiresorptive or anabolic treatments of osteoporosis
4. A liver function test or creatinine level above the normal range, or any other history suggesting liver or kidney disease to be confirmed by baseline screening
5. Incidence of diabetes mellitus by using the questionnaire and baseline screening
6. Participants with an estimated BMD T score <−2.5 or fragility fracture in the previous 6 months
7. Antibiotic intake in the previous 6 months
8. Smoking and intake of alcohol >2 units per day

The following criteria are to be confirmed by the baseline questionnaire:

1. Participants’ intake ability and allergic reactions to probiotic and prebiotic supplements
2. Intake of multivitamins and mineral supplements (prescribed or over the counter), antibiotics, or use of any other medication known to affect bone metabolism and/or gut microbiota
3. Presence of any systemic disease
4. Use of any medications such as HRT, glucocorticoids, estrogen, systemic cortisone, bisphosphonates, diuretics, antibiotics (for the gut microbiota), or other steroid hormones
5. Active physical activity, that is, ≥60 minutes of vigorous or moderate activity for ≥3 days.

### The Intervention: COPES-4-Bones Clinical Study

The study design is a randomized controlled trial (RCT). All the volunteer participants in the trial will undergo the following.

#### Health Questionnaire and Health Screening

##### Blood Test for the Initial Baseline Screening

Fasting blood samples will be collected at baseline for (bone biomarkers and inflammatory cytokines) routine laboratory tests as well as medical examinations to ensure that the participants are in good health. Abnormal results from this trial will be recommended for discussion with their doctors.

##### Initial Anthropometry at Baseline

The body weight of participants will be measured using a weight scale to the nearest 0.1 kg, and standing height will be measured using a stadiometer to the nearest 0.1 cm wearing light clothes and no shoes. BMI will be calculated as weight divided by height squared (kg/m²). Waist to hip ratio will be determined by measuring the waist and hip circumference to the nearest 0.1 cm using a nonstretchable tape. Other body composition measurements will be analyzed with DXA.

##### Baseline Questionnaire

The baseline questionnaire will include the following: sociodemographic, activity index and level, medications taken in the last 6 months, smoking status, and alcohol intake.

#### For Inclusion

##### Diet or Food Records

The dietary assessment of the intake of fermented milk and dairy products, including total energy, protein, minerals, and vitamin D, will be based on a 3-day diet diary. Face-to-face interviews by the principal investigator will ascertain the food record.

##### Venous Fasting

Blood, fecal, and urine samples will be collected at baseline and week 12. Blood will be collected by a phlebotomist between 8 and 10 AM after 12 hours of fasting (overnight). Table 1 shows all the variables that will be measured, rationale, and the methods to be used.

1. Fasting blood samples: blood samples will be collected at baseline and week 12, the end of the study, for the following:
  Bone metabolism markers: concentrations of serum or plasma total osteocalcin, CTx-I, total P1NP, and 25-hydroxyvitamin D will be measured using immunoassay kits and Roche Elecsys.
  Cartilage degradation markers: COMP precursor and CTx-II from serum will be measured.
  Parathyroid hormone and lipid profile tests will be measured using immunoassay kits.
  Inflammation markers: concentrations of inflammatory cytokines by BioLegend LEGENDplex Multi-Analyte and hs-CRP (high-sensitivity C-reactive protein) will be measured.
2. Spot urine samples: samples of a midstream urine specimen voided spontaneously by the participants will be collected at baseline and at the end of the study and tested for protein, creatinine, and electrolyte content as well as CTx-II by ELISA (enzyme-linked immunosorbent assay).
3. Fecal samples: samples will be collected at baseline and at the end of the study (week 12). The total genomic DNA will be extracted, and 16s ribosomal DNA will be amplified, prepped, and sequenced.

**Table 1:** Study outcome measures and rationale for use.

Variables		Rationale	Methods	Baseline	Week 12
**Blood analyses**					
	OC^a^	Bone formation markers	Electrochemiluminescence immunoassay using the Roche COBAS e411 system (Roche Diagnostics)	✓^b^	✓
	P1NP^c^	Bone formation markers	Electrochemiluminescence immunoassay using the Roche COBAS e411 system (Roche Diagnostics)	✓	✓
	CTx^d^-I	Bone resorption marker	ELISA^e^	✓	✓
	25(OH)D^f^	Serum Vit D to determine the amount of circulating vitamin	Isotope-dilution liquid chromatography-tandem mass spectrometry	✓	✓
	COMP^g^	Cartilage degradation markers	ELISA	✓	✓
	CTx-II	Cartilage degradation markers	ELISA	✓	✓
	PTH^h^	To assess the regulation of serum calcium concentration	Electrochemiluminescence immunoassay using the Roche COBAS e411 system (Roche Diagnostics)	✓	✓
	Lipid profile	To assess the lipid profile	Electrochemiluminescence immunoassay using the Roche COBAS e411 system (Roche Diagnostics)	✓	✓
	Inflammatory cytokines	To assess the inflammatory status	BioLegend LEGENDplex Multi-Analyte	✓	✓
	hs-CRP^i^	To assess the inflammatory status	Electrochemiluminescence immunoassay	✓	✓
Gut microbiota data		To determine changes in the bacterial community	16s ribosomal DNA	✓	✓
Diet diary		To obtain dietary intake data	3-day diet diary	✓	✓
Exercise history record (IPAQ^j^)		For initial randomization	IPAQ	✓	✓
Baseline questionnaire (sociodemographic and medication history record)		To obtain sociodemographic status and history	Questionnaires	✓	✓
Wearable fitness tracker record		To obtain exercise regime data	Wearable fitness tracker	✓	✓
Adherence to synbiotic supplement and exercise		Documentation of unused supplements or prescribed exercise session attendance	Record keeping	✓	✓
Anthropometry		Weight, height, and waist circumference measured by a researcher at Massey University	Tanita electronic scale and stadiometer	✓	✓
DXA^k^		BMD at the total hip, femoral, neck and spine (L1-L4) and body composition	DXA using Hologic QDR series Discovery A, Bone densitometer, and Apex system software version 4.5.3	✓	✓

^a^OC: osteocalcin.

^b^Variable accessed.

^c^P1NP: procollagen type 1 N-terminal propeptide.

^d^CTx: cross-linked C-telopeptide.

^e^ELISA: enzyme-linked immunosorbent assay.

^f^25(OH)D: 25-hydroxyvitamin D.

^g^COMP: cartilage oligomeric matrix protein.

^h^PTH: parathyroid hormone.

^i^hs-CRP: high-sensitivity C-reactive protein.

^j^IPAQ: International Physical Activity Questionnaire.

^k^DXA: dual-energy x-ray absorptiometry.

##### DXA Measurements

Body composition, bone mineral content, BMD, and T scores of the femoral neck, lumbar spine, and hip will be measured in participants at baseline screening.

All exercise intervention and synbiotic+exercise groups or participants will be given a wearable fitness tracker to wear during the exercise (brisk walking) of 10,000 steps. The Borg Rating of Perceived Exertion will be used to calculate intensity. Table 2 shows the interventions and dosage per participant.

**Table 2:** Intervention and dose administered to participants in each group.

Intervention and treatment groups	Description	Daily intake per day
Synbiotic	Probiotic supplement Prebiotic supplement	10 billion colony forming units of *Lactobacillus sp.* 8 grams of prebiotic fiber (inulin)
Exercise	Weight-bearing exercise	10,000 steps
Placebo	Placebo	Placebo with maltodextrin

### Statistical Analyses

G*Power statistical software version 3.1.9.7 developed in Heinrich Heine University Düsseldorf was used to calculate the sample size to ensure 95% CI with an α value of .05. We require 36 participants for each group. The within-subject SD for the primary outcome variables CTx-I and P1NP with a correlation of 0.5 was used. The number of volunteers was increased to 40 to allow for a dropout rate of 10%.

The results will be presented as either percentages or mean differences. Normality tests will be assessed through Shapiro-Wilk tests carried out on each parameter before analysis. The conventional analysis of variance (ANOVA) for RCT analysis will be used, and ANOVA for repeated measures will be applied to study treatment differences, period effect, and the interaction between treatment and period (carryover effect). IBM SPSS version 25 and Minitab statistical software version 19 (Minitab LLC) will be used for statistical analyses. Comparing groups' pretest with Mann–Whitney U test and then comparing pre- and postintervention results with Wilcoxon is one option and transforming data into ranks and performing an analysis of covariance (or ANOVA) is another option. All analyses will be considered statistically significant at *P*≤.05.

## Results

### Ethics and Collection of Data

Ethical approval for this study has been received from the Health and Disability Ethics Committee of New Zealand. The recruitment and collection of data will begin in September 2021. We aim to complete data collection by June 2022. Statistical analyses, report writing, and dissemination of results are expected to be completed by February 2023. Funding is being sought for the study.

### Expected Benefits or Outcomes

The anticipated outcomes of this study are a reduction in bone turnover as measured using CTx-I as a marker, as well as a reduction in inflammation (reduced or changed levels of specific cytokines such as hs-CRP, IL-6, and TNF-α).

With menopause and the loss of estrogen, bone turnover (formation and resorption) increases significantly. Over time, because of increased levels of IL-6 and inflammation, bone resorption (breakdown) overtakes bone formation, and this increase in bone resorption can be measured using the marker CTx-I. Several studies over the past 20 years have shown that a reduction in CTx-I is associated with long-term changes in bone density and a reduction in fractures. CTx-I most sensitively reflects the change in bone resorption after mineral supplementation or increased absorption of calcium and can predict the rate of bone loss and fracture risk in postmenopausal women. CTx-I may reflect parameters of bone strength unrelated to BMD, such as microarchitectural deterioration of bone tissue resulting in microcracks that act as stress risers or trabecular perforation.

Supplementation with a synbiotic will modify the gut microbiota and improve calcium and magnesium absorption. Prebiotic supplementation promotes bacterial growth that induces nondigestible carbohydrate fermentation, increases short-chain fatty acids (SCFAs), and reduces the pH of the gut, thereby increasing calcium absorption. The reduction in pH promotes the growth of bacteria that are less likely to cause inflammation, and SCFAs stimulate the effects of anti-inflammatory cytokines. Probiotic supplementation by *Lactobacillus* and *Bacillus* species may promote an immunoprotective response in the gut mucosa by reducing the levels of systemic inflammatory cytokines and preventing the reduction in bone density.

## Discussion

### Principal Findings

Degeneration of bone health in the form of osteoporosis, osteoporotic fractures, and osteoarthritis are major health care issues leading to a significant increase in morbidity and mortality in New Zealand and all over the world. Similarly, the growing number of patients with osteoporosis or osteoarthritis results in huge health care costs. The aim is to measure the effect of synbiotics, weight-bearing exercises (10,000 brisk walking steps per day), or a combination of both on gut microbiota, inflammation status, and bone health. This study is important for several reasons. This study is directed toward postmenopausal women who have experienced a rapid phase of bone loss 5 years postmenopause. The use of a synbiotic (a combination of probiotic and prebiotic) supplement is particularly novel in the modulation of the immune system, gut microbiota, and anti-inflammatory response. Studies are needed to measure the effects of synbiotic supplementation and weight-bearing exercise on gut microbiota, inflammatory status, and bone health. Randomization by exercise history will help eliminate the effects of the previous exercise regime for the study. There is a critical need for measures of bone turnover and not BMD only as a primary outcome.

The effects of probiotic or synbiotic supplementation on markers of inflammation have also been reported. A reduction in proinflammatory cytokines (eg, IL-1β, TNF-α, IL-6, and IL-8) and an increase in anti-inflammatory cytokines (IL-10 and IL-4) [44] were observed in response to these interventions. Similarly, studies have indicated the protective factors of probiotic supplementation [45] and exercise in bone metabolism and health [34]. Long-term participation in a relevant and targeted exercise regime is known to improve bone mechanical properties over time [46].

### Synbiotics: Mechanism of Action

Prebiotic supplementation promotes bacterial growth that induces nondigestible carbohydrate fermentation, increases SCFAs, and reduces the pH of the gut contents, thereby increasing calcium absorption. The reduction in pH promotes the growth of bacteria that are less likely to cause inflammation, and SCFAs stimulate the effects of anti-inflammatory cytokines. In addition, probiotic supplementation with *Lactobacillus* and *Bacillus* species promotes immunoprotective response or effects in the gut, reducing inflammatory cytokines and preventing the reduction in bone density [47].

### Markers of Bone Turnover

Prediction of bone loss and risk of fractures is conducted using biomarkers of bone turnover independent of bone density in women. The occurrence of menopause results in a period of bone loss, where the rate of bone resorption exceeds that of formation (approximately 5 years). A bone remodeling cycle of formation and resorption takes place between 4 and 6 months, replenishing approximately 5%-15% of the total bone mass in a year [46]. These data provide the rationale for the length of time and a comprehensive investigation of body composition and bone turnover status in the study described here.

The strength of the RCT will account for exercise history and the sample size and duration to ensure an adequately powered sample needed to detect clinically and statistically significant results.

### Ethics and Dissemination

#### Ethics

The study received ethical approval from the Health and Disability Ethics Committee of New Zealand.

#### Safety and Data Monitoring

The principal investigator and team will monitor the conduct, safety, and scientific integrity of the proposed clinical trial. Routine laboratory measurements, including liver and kidney function tests, blood glucose (nonfasting), and lipid profile (triglyceride, total cholesterol, high-density lipoprotein cholesterol, and low-density lipoprotein cholesterol) tests, will be performed at baseline and 12 weeks. Individuals with results that are outside the clinical range will be contacted and referred to their general practitioner. The safety precaution will be to inform all participants to report any effects of treatment, and in the unlikely event that 20% of the participants report severe diarrhea, a discontinuation of the study will be triggered. Each report will include the monitoring of compliance with informed consent and eligibility requirements, compliance with the recruitment plan according to protocol, follow-up data collection according to the protocol, expected and actual accrual, protocol violations, and patient withdrawals from the study.

#### Dissemination

A lay summary of the report will be communicated to all study participants. The results of the study will also be disseminated at various seminar presentations and feedback sessions at the College of Health, School of Health Sciences, Massey University, Palmerston North, New Zealand, and in manuscripts that will be submitted to a peer-reviewed journal.

### Strengths and Limitations of This Study

This study was designed as an RCT to account for exercise history, and the sample size and duration have been selected to ensure an adequately powered sample needed to detect clinically and statistically significant results. In this study, investigating the effects of both prebiotics and probiotics with and without weight-bearing exercise provides strong evidence for an RCT. The limitation of the study lies in the inability to extend the duration in terms of further follow-up.

### Conclusions

Our research study aims to decrease the possibility of osteoporotic fractures resulting because of the incidence of inflammation and loss of bone mass by improving body composition (lean and fat mass) among postmenopausal women after ≥5 years. This study compares the effectiveness of synbiotic supplementation and weight-bearing exercise intervention, both of which may be used as a therapy for bone health maintenance in postmenopausal women. To the best of our knowledge, this will be the first RCT to analyze the effects of both a synbiotic supplement and an exercise intervention in postmenopausal women. On the basis of the results obtained, a combination of synbiotic supplementation and exercise might serve as a noninvasive approach to manage and/or improve body composition and bone health in postmenopausal women.

## Abbreviations

ANOVA: analysis of variance
BMD: bone mineral density
COMP: cartilage oligomeric matrix protein
CTx-I: cross-linked C-telopeptide of type 1 collagen
DXA: dual-energy x-ray absorptiometry
ELISA: enzyme-linked immunosorbent assay
HRT: hormone replacement therapy
hs-CRP: high-sensitivity C-reactive protein
IL: interleukin
P1NP: procollagen type 1 N-terminal propeptide
QALY: quality-adjusted life-year
RCT: randomized controlled trial
SCFA: short-chain fatty acid
TNF: tumor necrosis factor
